# Poly-ether-ether-ketone wear particles induce a pro-inflammatory phenotype in a human monocytic cell line

**DOI:** 10.3389/fbioe.2025.1507248

**Published:** 2025-08-05

**Authors:** Shannon Jamieson, Philip Hyde, Patrick Card, David Deehan, John Kirby, Alison Tyson-Capper

**Affiliations:** ^1^ Translational and Clinical Research Institute, Newcastle University, Newcastle upon Tyne, United Kingdom; ^2^ School of Engineering, Newcastle University, Newcastle upon Tyne, United Kingdom; ^3^ Orthopaedics, Newcastle upon Tyne Hospitals NHS Foundation Trust, Newcastle upon Tyne, United Kingdom

**Keywords:** chemokines, macrophages, immunology, osteoarthritis, immune response

## Abstract

**Objectives:**

The aim of this study was to assess potential pro-inflammatory responses induced in a human monocyte cell line by poly-ether-ether-ketone (PEEK) particles. Investigations also focussed on the role of toll-like receptor 4 (TLR4) and reactive oxygen species (ROS) in immune responses to PEEK.

**Methods:**

PEEK particles were generated using a four-station multi-directional pin-on-plate wear simulator and used to treat THP-1 macrophages for 24 h at dosages of 0.5–50 μm^3^ per cell. THP-1 cell supernatant was used for protein secretion analysis using ELISA and gene expression investigations using RT-qPCR. TLR4 inhibition was also achieved using CLI-095 by treating cells prior to PEEK exposure. ROS production was investigated following PEEK treatment. IL-1β secretion was investigated by treating PEEK-exposed cells to 5 mM ATP for 1 h in order to assess the role of the inflammasome.

**Results:**

PEEK particles were not immediately cytotoxic to THP-1 macrophages and induced a significant increase in gene expression and protein secretion of IL-8, CCL2, CCL3, and CCL4 at the highest dose (p < 0.0001). This increase in pro-inflammatory genes and proteins was not inhibited following blockade of TLR4. ROS production was significantly upregulated in the PEEK-treated cells and IL-1β secretion was also significantly increased following the addition of ATP to PEEK-exposed THP-1 cells.

**Conclusion:**

PEEK particles are capable of inducing a pro-inflammatory phenotype in a human macrophage cell line which is not a result of TLR4 activation. PEEK particles do act in a PAMP-like manner and are able to induce ROS production as well as initiate inflammasome activation.

## Introduction

Total joint replacement (TJR) is the leading treatment option for end-stage osteoarthritis (OA) and involves replacement of diseased cartilage and bone with a prosthetic implant. Total knee replacement (TKR) is the most common type of joint replacement with close to 100,000 primary surgeries occurring per year in the United Kingdom (National Joint Registry, 2020). TKR implants are comprised of a femoral component and a tibial component with a spacer in the middle to allow for hinge movement similar to that of a healthy knee joint. By restoring a full range of movement, TKR aims to contribute to a greater quality-of-life for patients by allowing them to return to their normal day-to-day activities while alleviating OA symptoms such as pain and immobility. However, the hinge movement of metal components on the polyethylene spacer over time can result in material wear and the release of particulate debris into the peri-implant space. This polyethylene wear debris has been linked to osteolysis and additional bone breakdown, resulting in aseptic loosening and the need for revision surgery (Kobayashi et al., 1997). Revision surgery has a higher risk than primary TKR surgery, with outcomes including increased rates of venous thromboembolism, infection, and mortality (Badarudeen et al., 2017). Additionally, the cost of TKR revision surgery to the NHS is estimated as close to £10 million per annum (Kassam et al., 2012). These issues with revision surgery highlight the need for a low-wearing, longer lasting and biocompatible implant material which will be less susceptible to aseptic loosening in the long-term.

Recent studies have evaluated the biological impact of TJR wear debris by assessing the changes in pro-inflammatory cytokines such as IL-8, CCL2, CCL3, and CCL4 in patients with TKR complications as well as in immortalised human cell lines. Investigations of specific orthopaedic biomaterials such as ceramic oxides have demonstrated the ability of a human macrophage cell line to engulf the material leading to upregulation of the same inflammatory cytokines (Jamieson et al., 2021). Moreover, elevated levels of TNFα and IL-4 have been noted in an aseptic loosening cohort of patients (Jämsen et al., 2017). Additionally, polyethylene particles have been shown to induce IL-6 and IL-β secretion *in vitro* as well as cell death *in vivo* (Green et al., 1998). These findings confirm the importance of cytokine profiles in the response to orthopaedic biomaterials to identify which immune pathways are activated in aseptic loosening. By identifying the role of specific cytokines in inflammatory responses to TJR materials this can aid the development of biocompatible materials as well as pharmacological targeting of the immune system.

Poly-ether-ether-ketone (PEEK) is an aromatic polymer with a high melting temperature of 335°C and low solubility in solvents (Kurtz, 2012). PEEK was introduced as a biomaterial in the early 1990s predominantly for use in spinal fusion cages with high success rates due to good biocompatibility and stiffness of the material. The inherent properties of PEEK that make it an attractive option for orthopaedic indications include a Young’s modulus of ∼4GPa, which is similar to that of cortical bone (∼18 GPa). A mismatch in the Young’s modulus of a patient’s joint and an implant may lead to the misplacement of stress and force resulting in a phenomenon known as ‘stress-shielding-induced bone loss’. This slows the ongoing bone regeneration process meaning there is substantial bone loss and can contribute to osteolysis (Sumner, 2015) therefore in turn contributing to aseptic loosening and the need for costly revision surgery. Interestingly, in 2021, Invibio Ltd. reported the first primary TKR using a PEEK femoral component as part of an ongoing clinical trial, however, no study readout has since been reported. It is essential to assess the potential immunomodulatory effects of PEEK if it is to become a viable option for orthopaedic implants to ensure positive patient outcomes in the future with minimal immunomodulatory effects.

Mechanistic investigations of immune responses to biomaterials have focussed on activation of specific receptors such as toll-like receptor 4 (TLR4) and the nod-like receptor pyrin-containing domain 3 (NLRP3) inflammasome. Studies have demonstrated the ability of cobalt and ceramics to induce inflammatory responses and inhibition of TLR4 proven effective in ameliorating responses in human cell lines (Jamieson et al., 2021; Lawrence et al., 2016a). One study reported that TLR4-deficient mice implanted with polyethylene discs had an altered leukocyte adhesion profile compared to mice which were TLR4 positive, suggesting a role for TLR4 in the inflammatory profile to polymers (Rogers and Babensee, 2010). Additionally, reports which investigated the role of the NLRP3 inflammasome have demonstrated the ability of a cobalt-chromium, a common metal used in joint replacement, to increase secretion of IL-1β in a human macrophage model (Feng et al., 2020). It is therefore important to consider the potential role of TLR4 and NLRP3 in any pro-inflammatory responses observed following treatment with PEEK particles in order to compare PEEK biocompatibility to that of materials already widely used in TJR.

Due to the osteolytic nature of polymer wear debris currently used in TKR and the accumulating evidence of inflammatory effects of metal and ceramic biomaterials there is a need to address the paucity in the literature regarding potential pro-inflammatory immune responses that PEEK particles may generate. Human macrophages have been the focus of studies investigating the pro-inflammatory effects of biomaterials such as ceramic oxide nanopowders (Jamieson et al., 2021) and cobalt-chromium particles (Jamieson et al., 2023) previously due to their highly phagocytic nature and the ability to effectively inhibit TLR4. Therefore, the aim of this study was to utilise established methodologies to investigate the cytotoxic and inflammatory effects of PEEK particles on a human macrophage cell line in order to determine whether a similar effect was observed when compared to other biomaterials.

## Methods and materials

### PEEK particle generation

PEEK-OPTIMA™ LT1 rods and plates were supplied by Invibio Ltd. and cut to custom sizes to fit a four-station pin-on-plate (PoP) wear simulator (School of Engineering, Newcastle University). Experimental set up for the PoP rig is detailed in Supplementary Figure S1. Pins and plates were pre-soaked in deionised water for a minimum of 30 days and dried for 48 h immediately prior to debris generation in accordance with a protocol optimised by Chamberlain et al. (2019). 20 mL of deionised water was added to each station of the PoP wear simulator with plates and pins secured in place. The motors were then set to a speed of 1 Hz with a stroke length of 30 mm. Each pin had constant 360° rotation and a load of 40 N applied to provide 5.7 MPa of contact pressure. The PoP wear simulator was run for 1 million cycles based on ISO 17853 (ISO, 2011). PEEK wear particles were collected and gravimetric analysis carried according to ASTM F1714 guidelines (ASTM, 2018). Particle stock solutions were then subjected to gamma irradiation using a Gammacell 1,000 irradiator with a [137Cs] source (Nordion International Inc., Ottawa, Canada) at a rate of approximately 3.08 Gy/min in accordance with a protocol which was optimised by Dr Kathryn Chamberlain (Newcastle University). Sterilisation was confirmed through culture on agar plates. A 1 mg.mL^−1^ stock of PEEK particles used for vacuum filtration and assessment of the particle charge. 1 mL samples of the PEEK particles were used in conjunction with a Litesizer DLS 700 Particle Size Analyser (Anton Paar, Graz, Austria) and Kalliope Professional Software version 3.12.1 in order to determine the charge of the particles (Supplementary Figure S2). The PEEK stock was also diluted in 5 mL sterile water and sonicated for 30 min before sequential vacuum filtration through Cyclopore polycarbonate filter members (Whatman International, Buckinghamshire, United Kingdom) was carried out. Pore sizes used for filtration were 5 μm, 0.4 μm, and 0.015 μm. Filter membranes were then processed by EM Research Services (Newcastle University) and imaged via scanning electron microscopy (SEM) using a Tescan Vega 3LMU (Tescan, Brno, Czechia) at 8.5 kV. Images were taken at magnifications between x 5K and x 15K. Smaller working stocks of 0.5 mg.mL^−1^ were made by dilution of the particle suspension at a ratio of 1:1 in blank cell culture medium and stored at −20°C to help avoid repeated freeze-thawing and particle aggregation. Following SEM, ImageJ was used to characterise identified particles according to size by converting images into 8-bit and creating a binary image to visualise particles only. Once the binary image was created, manual thresholding was used separate particles from background and set to 243–254. Individual particles were then manually drawn around and overlapping particles were excluded in order to ensure that only particles where their entire circumference was observable were measured. Therefore, the particle size was determined using their diameter. Size of particles was then converted to a percentage by calculating the number of particles per size range investigated against the total number of particles observed.

### THP-1 cell line

THP-1 cells were used throughout this study as they are a model human monocytic cell line derived from acute monocytic leukaemia (ATCC TIB-202) (Chanput et al., 2013). Cells were cultured in RPMI-1640 medium supplemented with 10% foetal bovine serum, 50 U/mL penicillin, 50 µg/mL streptomycin, and 2 mM L-glutamine (Sigma-Aldrich, Gillingham, United Kingdom). Once 90%–100% confluent, cells were seeded at a density of 500,000 per well of a 12-well tissue culture plate (Greiner Bio-One, Kremsmunster, Austria). Cells were activated using 5 ng/mL phorbol 12-myristate 13-acetate (PMA) (Peprotech, London, United Kingdom) for 24 h followed by removal of cell culture media, gentle washing with PBS, and then addition of fresh media to rest the cells overnight prior to all subsequent assays.

### Cell treatments

PEEK particle stock solutions were thawed and sonicated for 10 min and used at 0.5–50 μm^3^ per cell dosages in conjunction with the PMA-differentiated THP-1 cell line. Volumetric dosing of PEEK particles was chosen as the particles are not soluble and this was based on previous biomaterial studies which used cobalt-chromium particles and ceramic oxide nanopowders to take into account the number of cells seeded as opposed to the volume of liquid per well (Jamieson et al., 2021; Mawdesley, 2020). TLR4-specific lipopolysaccharide (LPS) (Alexis Biochemicals, United States) was diluted in complete culture RPMI-1640 medium and used as a positive control at a working concentration of 10 ng/mL. Untreated cells (UT) were used as a negative control throughout.

### Cytotoxicity assays

THP-1 cells were differentiated with PMA and then treated with PEEK particles as described in the protocol above. 1 mL Accutase (Sigma-Aldrich) was added and cells incubated for 15 min to ensure full detachment from the tissue culture plastic. Cells were then collected and centrifuged at 5,500xg for 5 min until a pellet was formed. The supernatant was removed and the cell pellet resuspended in 200 μL complete cell culture medium. The cell suspension was then mixed with trypan blue (Sigma-Aldrich) at a ratio of 1:1.10 μL of the mixed solution was then pipetted onto a Luna cell counting slide and used with the Luna II (Logos Biosystems, Seoul, South Korea) fully automated cell counter. Percentage viability was then calculated. Furthermore, THP-1 cells were seeded into a 96-well tissue culture plate (Greiner Bio-One) at a density of 50,000 and differentiated with PMA prior to treatment with PEEK particles as described above. THP-1 cells were then used with the XTT Cell Proliferation Kit II (Sigma-Aldrich) according to the manufacturer’s instructions and absorbance read at 450 nm using a BioTek Synergy HT microplate reader. Cobalt chloride hexahydrate (CoCl_2_) (Sigma-Aldrich) was used as an inducer of cytotoxicity to ensure the sensitivity of the assay. CoCl_2_ was diluted in cell culture medium and cells were treated at a concentration of 1.5 mM in accordance with Lawrence et al. (2014).

### Quantitative real-time PCR (qRT-PCR)

The ReliaPrep™ RNA Miniprep System (Promega, Wisconsin, United States) was used for RNA isolation in accordance with the manufacturer’s instructions. cDNA was then synthesised from the isolated RNA using the Tetro cDNA Synthesis Kit (BIoLine Meridian Bioscience, Ohio, United States). qRT-PCR was carried out using Taqman gene expression probes (ThermoFisher). Four potential housekeeper genes were assessed by comparing cycle threshold (Ct) value following exposure to PEEK as detailed above. Due to a consistently low Ct value and minimal variation across PEEK treatments (Supplementary Figure S3) 18S was used as a housekeeper gene throughout subsequent analysis. Relative fold change was calculated using the 2^−ΔΔCT^ method.

### Enzyme-linked immunosorbent assay (ELISA)

Human ELISA DuoSet kits (R&D Systems, Minneapolis, United States) were used in accordance with the manufacturer’s instructions in order to quantify protein secretion from THP-1 cells following PEEK treatments. The proteins investigated were IL-8, CCL2, CCL3, CCL4, and IL-1β.

### TLR4 inhibition

CLI-095 (Invivogen, San Diego, United States) is a small-molecule inhibitor of TLR4 and was used to investigate the involvement of TLR4 in any pro-inflammatory responses to PEEK particles exhibited by the THP-1 cells. CLI-095 has been previously used to demonstrate the TLR4-specific inflammatory responses due to cobalt ions and ceramic oxide nanopowders (Jamieson et al., 2021; Lawrence et al., 2016a). Cells were pre-treated with CLI-095 at a concentration of 1 μg/mL for 6 h before being removed. Cells were then treated with the PEEK particles as described above.

### Reactive oxygen species (ROS) assay

ROS production was measured using the DCFDA Cellular ROS Detection Assay Kit (Abcam, Cambridge, United Kingdom) according to the manufacturer’s instructions. THP-1 cells were seeded at a density of 50,000 in a black clear-bottom 96-well tissue culture plate (Greiner Bio-One, Austria) and differentiated with PMA as described above. Culture media was removed and cells briefly washed in 100 μL 1X Buffer. The buffer was then replaced with 100 μL of diluted DCFDA solution and plates left to incubate at 37°C for 45 min in the dark. The DCFDA solution was then removed and 100 μL of 1X Supplemented Buffer was added briefly to the cells. The cells were then treated with PEEK particles as described above. Cells treated with 200 μM H_2_O_2_ were used to ensure that the assay procedure had worked. Plates were incubated for 6 h at 37°C in the dark and then fluorescence measured at Ex/Em 485/535 nm using a BioTek Synergy HT microplate reader.

### Inflammasome activation

THP-1 cells were seeded at a density of 500,000 cells per well of a 12-well tissue culture plate (Greiner Bio-One) and activated with PMA as described above. Following cell stimulation with LPS or PEEK particles for 23 h, adenosine 5′-triphospate disodium salt (ATP) (Invivogen) was added at a concentration of 5 mM for 1 h in order to induce IL-1β cleavage and secretion.

### Correlation analysis

Spearman’s rank correlation analysis was carried out in RStudio 4.4.0 to assess the relationship between protein secretion and relative gene expression for IL-8, CCL2, CCL3, and CCL4. UT and LPS samples were excluded from this analysis as they were control conditions. Each condition included n = 3. Correlation coefficients (R) were calculated and scaled from 1 to −1 for each PEEK dosage and a correlation matrix was then created. p-values were calculated using a t-distribution and significance was set at <0.05.

### Statistical analysis

Statistical analysis was performed with GraphPad Prism 9.5.0 using a one-way analysis of variance (ANOVA) with Dunnett’s multiple comparisons unless otherwise stated. All error bars represent standard error of the mean and all graphs are representative of three biological repeats (n = 3).

## Results

### PEEK particle characterisation

To evaluate the morphology of PEEK particles generated in this study and compare them to others generated using similar systems, isolated particles were subjected to sequential vacuum filtration and subsequent SEM. ImageJ was then used to determine particle size from SEM images. A DLS 700 Particle Size Analyser was used to determine PEEK particle charge.

**FIGURE 1:**
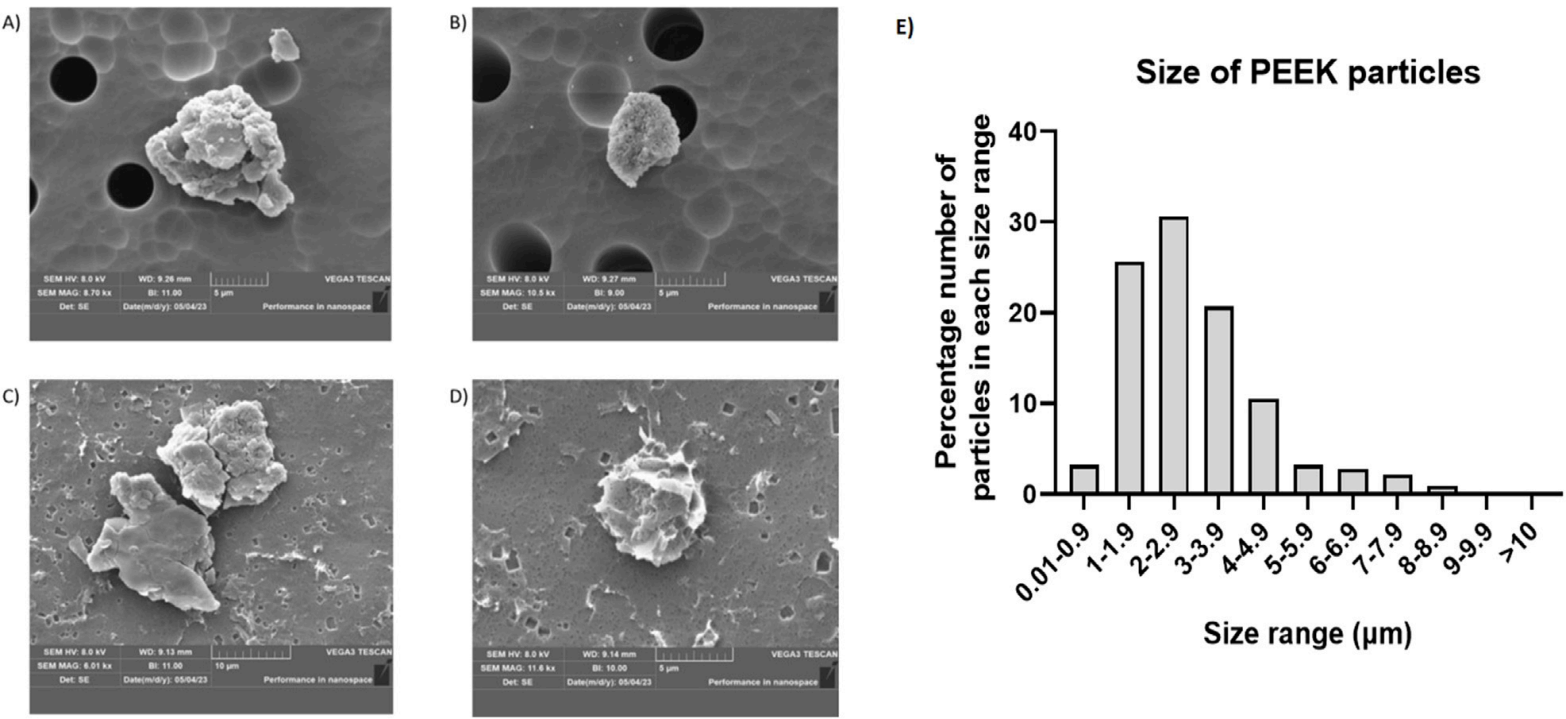
Example SEM images of PEEK particles and the corresponding size distribution. Following isolation of PEEK particles after generation using a four-station pin-on-plate simulator scanning electron microscopy was used to image individual particles. Representative images were captured at **(A)** x8.70K magnification, **(B)** x10.5K magnification, **(C)** x6.01K magnification, **(D)** x11.6K magnification with overall magnifications used ranging from x5.0K to x15.0K ImageJ was then used to perform particle size distribution analysis by creating binary images. Once 8-bit binary images were generated, thresholding was performed manually and set to 243–254. Approximately 150 particles were used for characterisation of each sample. **(E)** Particle size was then calculated against the total number of particles used for characterisation in order to give a percentage size distribution in order to gauge the relative size of PEEK particles generated. The majority of particles (87%) were in the 1–5 μm size range.

In the SEM images (Figures 1A–D), the PEEK particles observed were similar in morphology to those generated by Dr Kathryn Chamberlain (Newcastle University). There was a consistent shape as well as well-defined edges present, and representative images were captured at magnifications (A) x8.70K, (B) x10.5K, (C) x6.01K, (D) x11.6K. The overall charge of the PEEK particles was −22.3662 mV with a standard deviation of 0.49 based on three 1 mL aliquots at 100 runs per sample (Supplementary Figure S2).

Particle size was calculated as a percentage against total number of particles counted (∼150) following analysis using ImageJ and ∼87% of particles measured fit within the 1–5 μm size range (Figure 1E).

Overall, these data indicate the replicability of generating PEEK particles using a four-station multi-directional PoP wear simulator as well as demonstrating the negative zeta potential and size which may go on to influence phagocytosis.

### PEEK particles do not alter THP-1 viability or proliferation

To evaluate the potential cytotoxicity of PEEK particles, PMA-differentiated THP-1 cells were exposed to 0.5–50 μm^3^ per cell PEEK particles for 24 h. Cell viability was then assessed using trypan blue exclusion and proliferative capacity of the cells was measured using an XTT assay.

**FIGURE 2:**
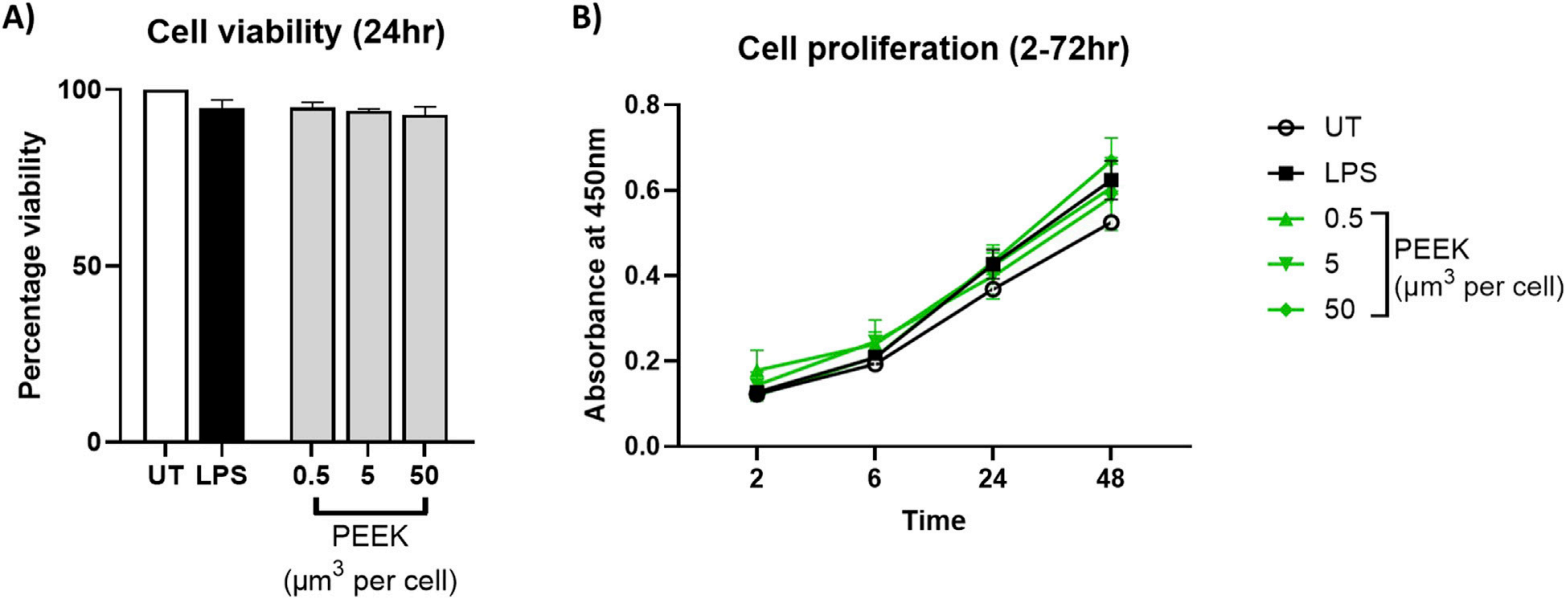
Cell viability following PEEK exposure. THP-1 cells were differentiated with PMA for 24 h and then rested overnight before being exposed to PEEK particles at a concentration of 0.5, 5, or 50 μm^3^ per cell for 24 h before cellular viability was assessed using trypan blue exclusion and XTT used for cellular proliferation investigations. **(A)** There was no significant change in the cell viability of PEEK-treated THP-1 cells at any dosage when compared to the untreated control (p = 0.0826). **(B)** There was no significant change in the cellular proliferation of THP-1 cells treated with PEEK particles at any dosage or time point when compared with the untreated controls (p = 0.9943). Graphs are representative of n = 3. One-way ANOVA with Dunnett’s multiple comparisons test was used to calculate statistical significance comparing treated samples to the untreated control.

There was no statistically significant difference in the percentage of viability cells when comparing PEEK treatments with the untreated control which can be seen in Figure 2A (p = 0.0826). Viability remained above 90% at all tested PEEK dosages.

Similarly, analysis of proliferative capacity using XTT (Figure 2B) showed no significant changes following exposure to PEEK particles at all dosages compared to untreated controls (p = 0.9897). Absorbance values remained stable across conditions therefore indicating no alteration in mitochondrial function.

Together, these data confirm that PEEK particles, at the tested concentrations and exposure duration, do not induce detectable cytotoxic effects in PMA-differentiated THP-1 cells.

### PEEK particles induce a pro-inflammatory phenotype in THP-1 cells

Other materials used for joint replacement implants have been shown to increase pro-inflammatory gene expression and protein secretion in PMA-differentiated THP-1 cells (Jamieson et al., 2021). Therefore, PMA-differentiated THP-1 cells were treated with increasing volumetric dosages of PEEK particles (0.5, 5, and 50 μm^3^ per cell) for 24 h to assess whether similar outcomes were observed. Relative gene expression was assessed using RT-qPCR and protein secretion was measured in cell supernatants by ELISA.

**FIGURE 3:**
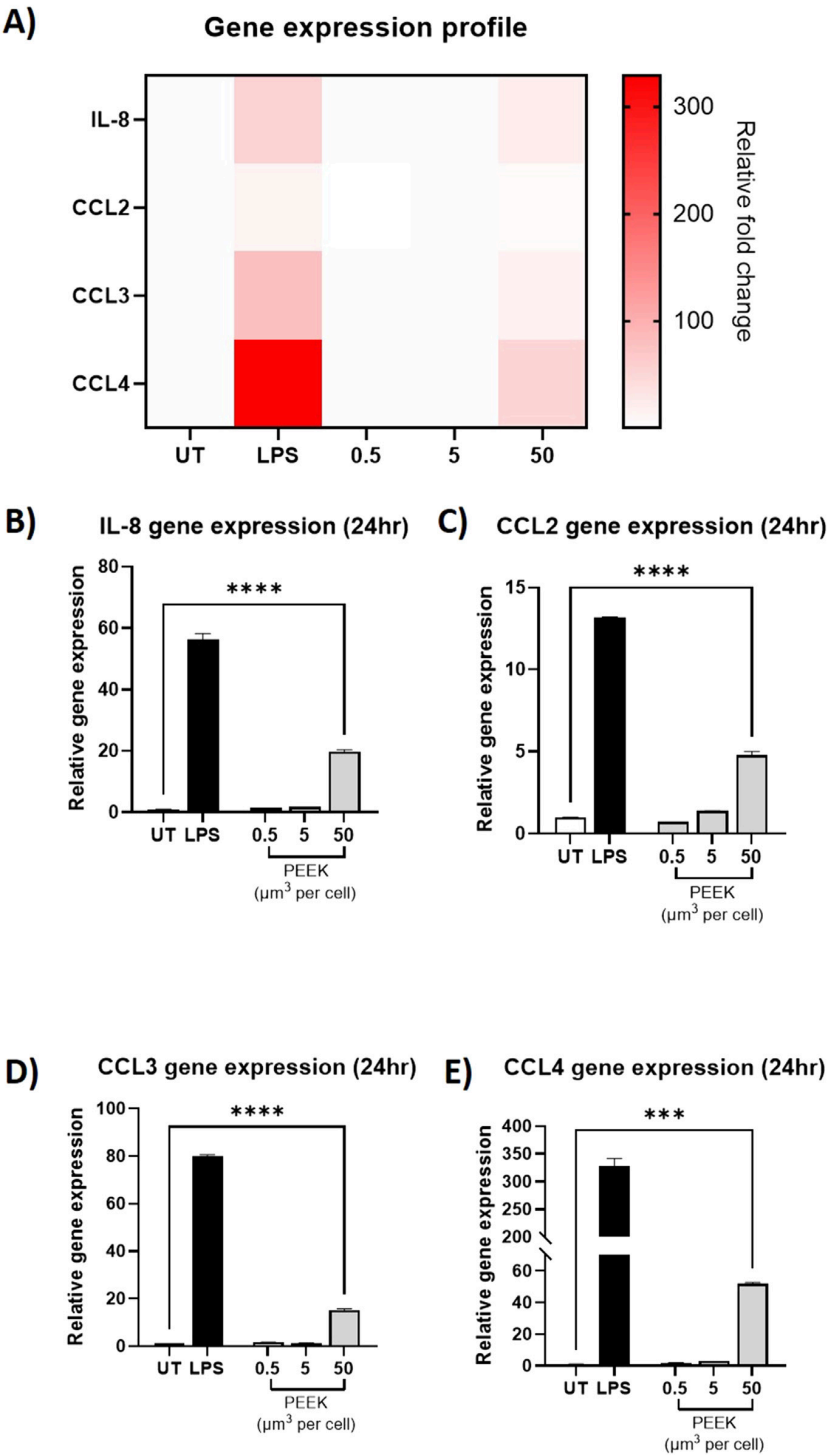
Relative gene expression following exposure to PEEK particles. THP-1 cells were treated with LPS (10 ng/mL) or 0.5–50 μm^3^ PEEK particles per cell for 24 h before RT-qPCR was used to assess the relative gene expression of *IL-8, CCL2, CCL3*, and *CCL4* compared to untreated cells (UT) using Taqman RT-qPCR. **(A)** A heatmap of relative fold change per treatment for *IL-8, CCL2, CCL3, and CCL4* was constructed to show the global inflammatory profile with respect to gene expression. As can be seen, gene expression relative fold changes were highest in LPS-treated or 50 μm^3^ PEEK-treated cells. THP-1 cells treated with 50 μm^3^ per cell PEEK particles experienced significant upregulation of **(B)** *IL-8* (p < 0.0001), **(C)** *CCL2* (p < 0.0001), **(D)** *CCL3* (p < 0.0001), *and* **(E)** *CCL4* (p < 0.0001). Each graph is representative of n = 3 and error bars represent the standard error of the mean. One-way ANOVA with Dunnett’s multiple comparisons test was used to calculate statistical significance comparing treated samples to the untreated control.

In the RT-qPCR analysis (Figure 3), there was a statistically significant increase in the relative gene expression of *IL-8, CCL2, CCL3, and CCL4* in the cells treated with 50 μm^3^ per cell PEEK particles when compared to the untreated control (all p < 0.0001).

**FIGURE 4:**
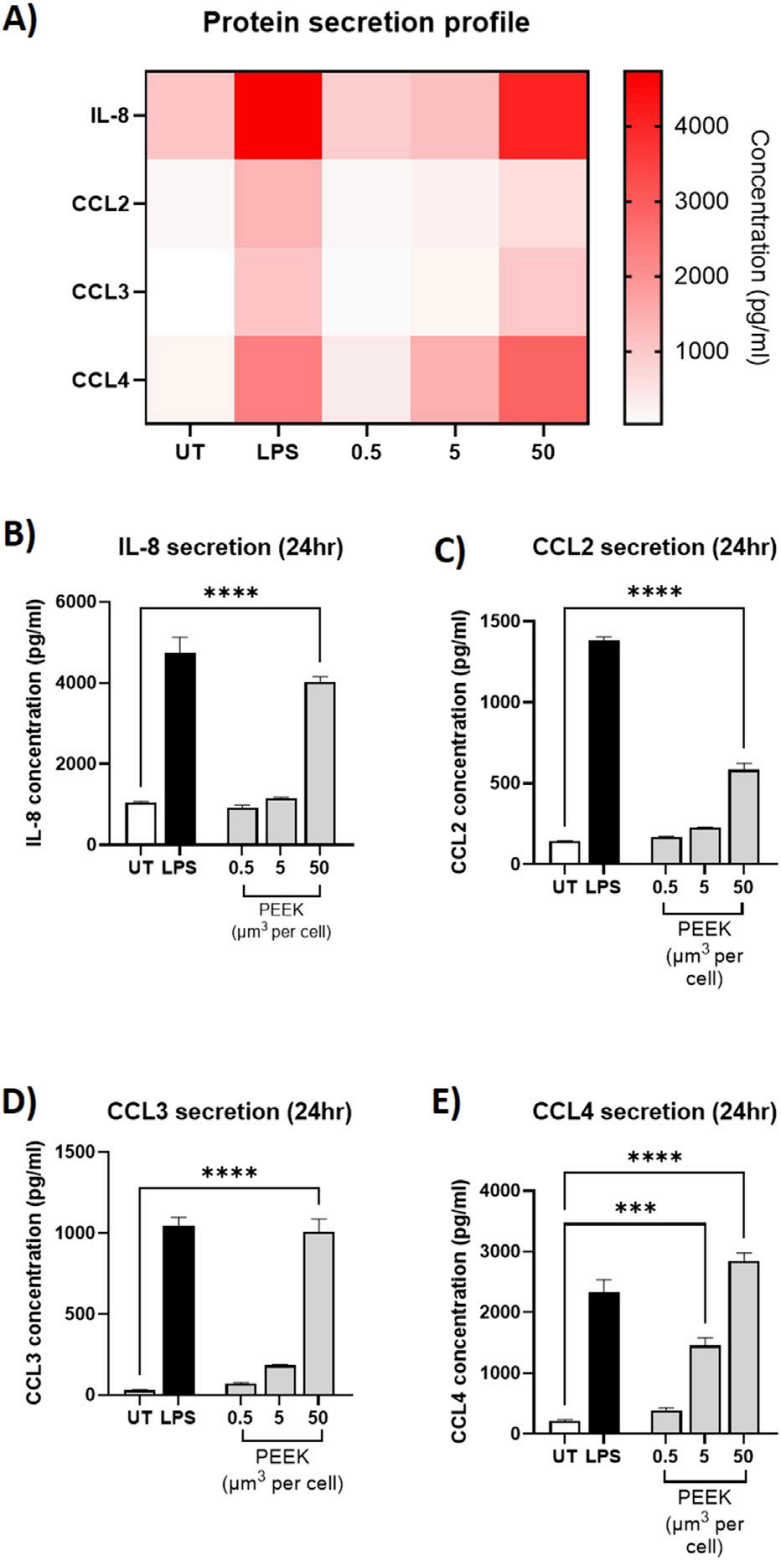
Protein secretion following exposure to PEEK particles. THP-1 cells were exposed to PEEK particles at 0.5, 5, or 50 μm3 per cell for 24 h. Supernatant was collected and used for protein secretion analysis of IL-8 CCL2, CCL3, and CCL4 using Human DuoSet ELISA kits purchased from R&D Systems. **(A)** A heatmap of protein concentration per treatment for *IL-8, CCL2, CCL3, and CCL4* was constructed to show the global inflammatory profile with respect to gene expression. As can be seen, gene expression relative fold changes were highest in LPS-treated or 50 μm^3^ PEEK-treated cells. THP-1 cells treated with 50 μm^3^ per cell PEEK particles experienced significant secretion of **(B)** IL-8 (p < 0.0001), **(C)** CCL2 (p < 0.0001), **(D)** CCL3 (p < 0.0001), and **(E)** CCL4 (p < 0.0001). Additionally the cells which received a 5 μm^3^ per cell dosage of PEEK particles also experienced a significant upregulation in CCL4 secretion (p = 0.0002). Each graph is representative of n = 3 and error bars represent the standard error of the mean. One-way ANOVA with Dunnett’s multiple comparisons test was used to calculate statistical significance comparing treated samples to the untreated control.

Similarly, analysis of protein secretion by ELISA (Figure 4) showed statistically significant increases in protein secretion of IL-8, CCL2, CCL3, and CCL4 from the cells exposed to the 50 μm^3^ per cell PEEK particles dosage (all p < 0.0001). Interestingly, there was also a statistically significant increase in CCL4 protein secretion at the intermediate 5 μm^3^ per cell dosage of PEEK particles (p = 0.0002).

Together, these data demonstrate the ability of PEEK particles, at 50 μm^3^ per cell with an exposure time of 24 h, to induce a pro-inflammatory phenotype in PMA-differentiated THP-1 cells as characterised by changes to gene expression and protein secretion.

### Pro-inflammatory gene expression and protein secretion positively correlate with increased PEEK dosage

To assess whether there was a relationship between the changes in relative gene expression and protein secretion observed in response to PEEK particles, Spearman’s rank correlation analysis was carried out.

**FIGURE 5:**
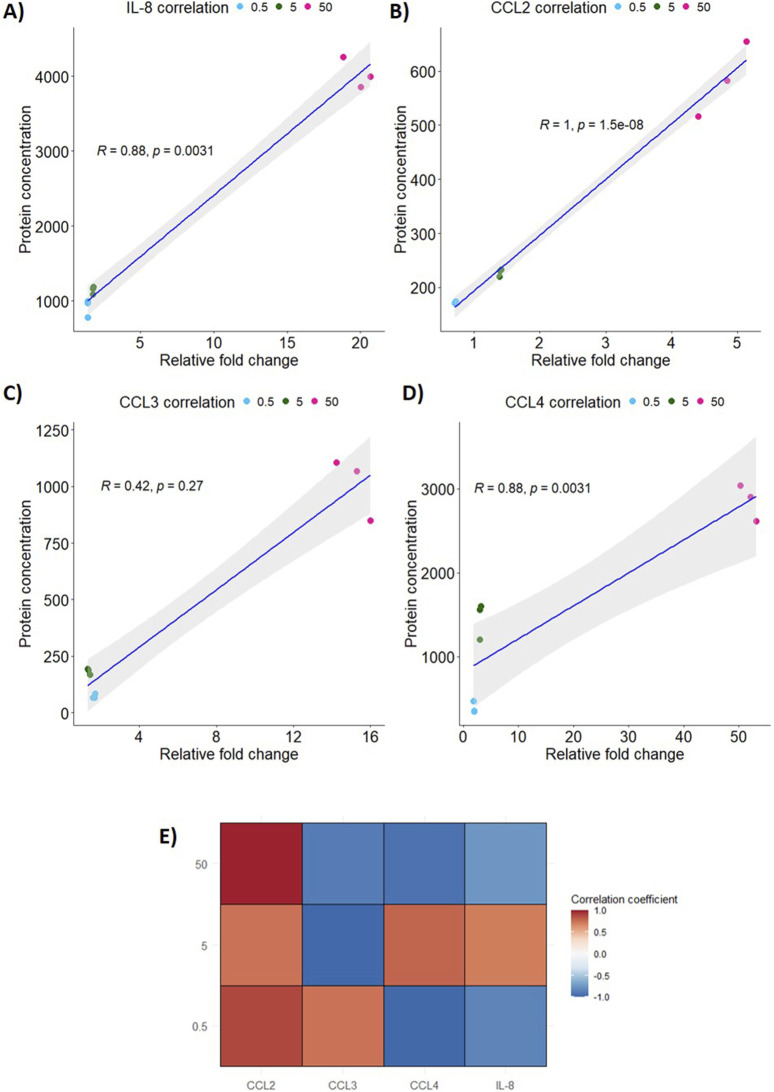
Correlation of relative gene expression to protein secretion in PEEK-treated THP-1 cells. Spearman’s rank correlation analysis was performed using RStudio 4.4.0 to analyse the relationship between protein secretion and gene expression following exposure to PEEK particles. **(A)** IL-8 expression and secretion was highly significantly positively correlated with an R of 0.88 (p = 0.0031). **(B)** CCL2 had the most significant positive correlation between protein secretion and gene expression (p < 0.0001) based on R = 1. **(C)** CCL3 was the marker assessed that did not have a significant positive correlation with R = 0.42 (p = 0.027). **(D)** CCL4 expression and secretion was significantly positively correlated (R = 0.88, p = 0.0031). **(E)** The global inflammatory profile was assessed through creation of a correlation matrix to assess the changes in gene expression and protein secretion for each PEEK dosage used. CCL2 had positive correlation coefficients at all dosages where CCL3 only had a positive correlation coefficient at the 0.5 μm^3^ per cell dosage. CCL4 and IL-8 has positive correlation coefficients at the 5 μm^3^ per cell PEEK particle dosage. All other dosages yielded negative correlation coefficients for CCL3, CCL4, and IL-8.

In the correlation analysis, both IL-8 (Figure 5A) and CCL4 (Figure 5D) has a statistically significant positive correlation of 0.88 (p = 0.0031). However, CCL3 (Figure 5C) has a weaker positive correlation of 0.42 which was not statistically significant (p = 0.27). CCL2 (Figure 5B) has the highest and most statistically significant correlation of all the inflammatory markers tested of 1 (p < 0.0001).

In order to analyse correlations at individual dosages of PEEK a correlation coefficient matrix was created (Figure 5E). CCL4 and IL-8 correlations were positive for the 5 μm^3^ per cell PEEK dosage and were negative for other PEEK concentrations. CCL3, however, did not have a positive correlation at 5–50 μm^3^ per cell PEEK and only at the 0.5 μm^3^ per cell dosage.

This data shows that changes to relative gene expression and protein secretion induced by PEEK, at the tested exposure duration, are positively correlated.

### TLR4 does not play a role in PEEK-mediate inflammatory responses

Due to the implication of TLR4 in the inflammatory responses to other biomaterials by PMA-differentiated macrophages, inhibition experiments were carried to determine whether the PEEK-induced inflammatory responses observed in this study occurred via the same mechanism. Inhibition of TLR4 was achieved through the use of CLI-095 for 6 h prior to PEEK exposure at the highest volumetric dose (50 μm^3^ per cell) for 24 h. Relative gene expression of *IL-8* and protein secretion of IL-8 were assessed using RT-qPCR and ELISA, respectively.

**FIGURE 6:**
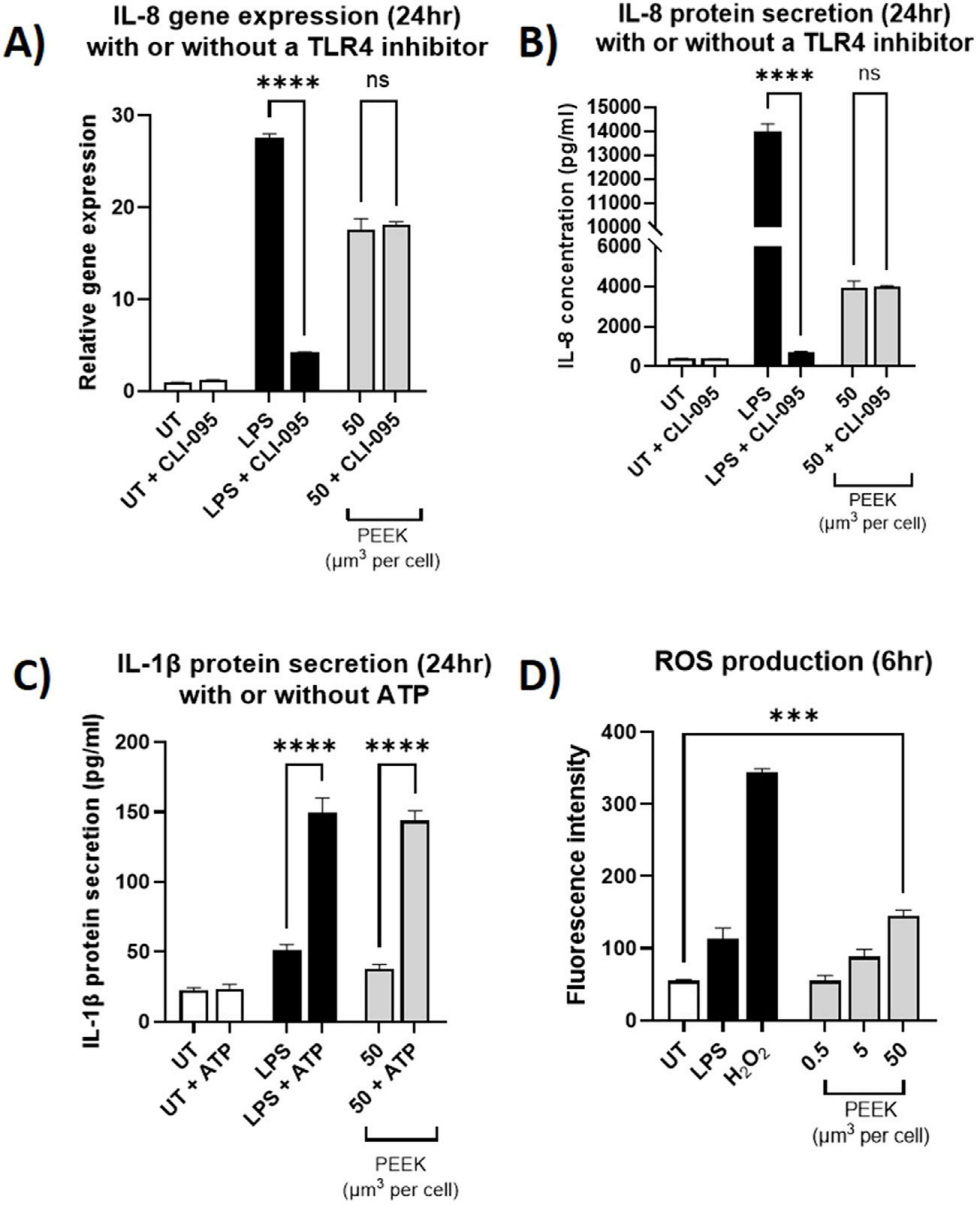
Investigating the inflammatory pathways activated by PEEK particles. THP-1 cells were differentiated using PMA and then pre-treated with CLI-095 for 6 h and before being exposed to LPS (10 ng/mL) or PEEK particles (50 μm^3^ per cell) for 24 h. Cells were collected and used for *IL-8* gene expression analysis by RT-qPCR. Supernatant was collected and used for ELISA analysis of IL-8 protein secretion. **(A)** There was no significant change in the *IL-8* gene expression in the THP-1 cells which received a pre-treatment with CLI-095 compared to those which did not prior to PEEK exposure (p = 0.9643). **(B)** The cells which received CLI-065 prior to PEEK exposure did not have a significant change in IL-8 protein secretion compared to those which did not receive TLR4 blockade (p > 0.9999). **(C)** THP-1 cells were treated with LPS (10 ng/mL) or PEEK particles (50 μm^3^ per cell) for 23 h before being exposed to ATP (5 mM) for 1 h. Cell supernatant was collected and used in conjunction with ELISA in order to determine IL-1β protein secretion. The cells which were exposed to PEEK and then ATP saw a significant increase in IL-1β secretion when compared to those which did not receive an ATP treatment (p < 0.0001). **(D)** Activated THP-1 cells were treated with DCFDA fluorescent dye prior to treatment with LPS (10 ng/mL), H_2_O_2_ (200 μM), or 0.5–50 μm^3^ per cell PEEK particles for 6 h. At the 50 μm^3^ per cell dosage there was a significant increase in ROS production (p = 0.0002) when compared to untreated cells. Each graph is representative of n = 3 and the error bars represent standard error of the mean. One-way ANOVA with Dunnett’s multiple comparisons test was used to calculate statistical significance comparing treated samples to the untreated control.

Following inhibition of TLR4 there was a statistically significant decrease in *IL-8* relative gene expression (Figure 6A) and IL-8 protein secretion (Figure 6B) in the PMA-differentiated THP-1 cells that received TLR4-specific LPS exposure (both p < 0.0001). This data indicates the ability of CLI-095 to effectively ameliorate TLR4 responses.

Conversely, no statistically significant difference was observed in the relative gene expression of *IL-8* following inhibition of TLR4 and subsequent PEEK exposure (Figure 6A) (p = 0.9643). Additionally, measurement of protein concentration (Figure 6B) revealed no statistically significant change in IL-8 secretion in the PMA-differentiated THP-1 cells pre-treated with CLI-095 and exposed to PEEK compared to those which received PEEK only (p > 0.9999).

Together, this data confirmed that PEEK particles, at the tested dosage and exposure time, do not induce inflammatory responses via TLR4 in PMA-differentiated THP-1 cells. Therefore, further mechanisms were investigated.

### PEEK particles induce ROS production and prime the inflammasome

After demonstrating that TLR4 was not involved in the responses to PEEK particles observed in this study, other mechanisms of immune cell activation were investigated. ROS production was assessed using a DCFDA ROS Detection Kit and inflammasome activation was investigated using the addition of ATP to PEEK-treated PMA-differentiated THP-1 cells. Cell supernatant was then used for ELISA to determine the secretion of IL-1β.

In the model of the inflammasome, (Figure 6C), there was a statistically significant increase in the secretion of IL-1β in the PMA-differentiated THP-1 cells which received LPS and ATP compared to LPS alone (p < 0.0001). This demonstrates the ability of LPS to prime PMA-differentiated THP-1 cells and the subsequent exposure of ATP to activate the cells. Similarly, in the cells which received 50 μm^3^ per cell PEEK particles there was a statistically significant increase in IL-1β secretion following the addition of ATP (p < 0.0001).

In the ROS detection assay (Figure 6D), there was statistically significant increase in ROS production in the PMA-differentiated THP-1 cells exposed to the highest volumetric dose of PEEK particles (50 μm^3^ per cell) (p = 0.0002). Fluorescence intensity was significantly increased when compared to untreated controls.

Together, these data demonstrate the ability of PEEK particles, at 50 μm^3^ per cell at the tested exposure times, to induce detectable increases in ROS production as well as the ability to prime PMA-differentiated THP-1 cells.

## Discussion

Studies investigating the cytotoxicity and biocompatibility of PEEK have focussed on whether it causes any adverse effects in its bulk form (Stratton-Powell et al., 2016). However, there have been few investigations of the potential cytotoxic effect of PEEK particles. Firstly, it was determined that the overall charge of the PEEK particles was −22.36 mV. Koner et al., recently showed the ability of THP-1 macrophages to change their morphology and shape following exposure to microplastics with a negative zeta potential, therefore suggesting a level of uptake being exhibited by the immune cells (Koner et al., 2023). Moreover, it has been noted that the presence of proteins in cell culture may also influence the uptake of particles by macrophages and therefore further investigations should aim to better establish the phagocytic profile of immune cells in response to PEEK particles. Before *in vitro* cell culture experiments were carried out, it was important to determine the effects of the PEEK particles on cell viability and proliferation as this has not been previously established. Results show there was no significant change in cell viability to PEEK particles. This provides strong evidence to confirm the biocompatibility and minimal cytotoxic effects of PEEK particles. These findings are also consistent with those of Howling et al., who found no significant cytotoxic effect of PEEK-OPTIMA particles on the L929 fibroblast cell line (Howling et al., 2004). Additionally, the proliferative capacity of the THP-1 cell line was not significantly affected when compared to untreated cells in all of the dosages (0.5, 5, 50 μm^3^ per cell) investigated. These findings are similar to those of Scotchford et al., who reported that PEEK did not have an impact on the attachment and proliferation of human osteoblast cells (Scotchford et al., 2003). Further investigations of PEEK-induced cytotoxicity should address gaps in this study pertaining to longer exposures as well as the potential to utilise specific markers such as Annexin V to provide a deeper understanding of cellular stress.

The data presented within this study demonstrates a shift in the global inflammatory profile of macrophages in response to PEEK particles therefore suggesting that PEEK may not be a suitable biocompatible implant material for orthopaedic indications. IL-8 is a known inducer of chemotactic neutrophil migration *in vivo* via CXCR1 and CXCR2 receptor activation, therefore making it an important component of the innate immune response to clear pathogens (Lassus et al., 2000). Studies by Koulouvaris et al., and Jamsen et al., demonstrated the link between increased IL-8 and the need for revision surgery due to aseptic loosening (Jämsen et al., 2017; Koulouvaris et al., 2008). The increased expression and secretion in response to PEEK particles suggests an important role of IL-8 in the pathogenesis of aseptic loosening and future studies should aim to further investigate which pathways are the driving producers of IL-8 in aseptic loosening. CCL2 is a chemoattractant specific for monocytes, T cells and NK cells and activates CCR2 and CCR4 receptors present on the cell surface. Therefore, an upregulation of CCL2 expression in response to a biomaterial is particularly important due to the ability of CCL2 to recruit both innate and adaptive immune cells. Du et al., reported high levels of CCL2 seen in immunohistochemical analysis of explanted tissue from a rabbit model that received a PEEK implant (Du et al., 2018). Interestingly, Dyskova et al., showed elevated levels of IL-8 and CCL2 in tissue from aseptic loosening patients when compared to non-aseptic loosening indications (Dyskova et al., 2019) and together these findings demonstrate their importance in the development of inflammatory responses to biomaterials including but not limited to PEEK. The gene expression and protein secretion of CCL3 was also significantly upregulated. CCL3 is similar to CCL2 in that it is a chemoattractant for monocytes but it is also known to induce osteoclast formation (Dapunt et al., 2014). This demonstrates the importance of CCL3 in inflammatory osteolysis, which is observed in aseptic loosening. Interestingly, Tomankova et al., reported that CCL3 mRNA was lower in aseptically failed total knee replacements compared to total hip replacements in a cohort of patients undergoing revision (Tomankova et al., 2014). This is important to note as these findings highlight differences in inflammatory responses depending on implant location. Future studies could therefore aim to stratify the immune response to implant materials based on location in order to gain a greater understanding of how aseptic loosening develops. CCL4 is a chemokine which has a strong binding affinity for CCR5 which is present on the cell surface of monocytes, therefore making it particularly important in early-stage innate immune responses. Patients experiencing chronic and unresolved inflammation with failed total knee replacement have elevated levels of CCL4 and other pro-inflammatory markers in their synovial fluid (Paish et al., 2019) and CCL4 has also been implicated in the inflammatory responses to ceramic oxide nanopowders in THP-1 macrophages (Jamieson et al., 2021). Further investigation of the role of CCL4 would be particularly interesting in future studies as it was the only chemokine which was elevated significantly at a lower dosage of PEEK particles in this study. The response observed at the intermediate dose of PEEK particles may be indicative of a key role of CCL4 in the response to biomaterials which has not yet been fully investigated within the literature.

Interestingly, correlation analysis between mRNA levels and protein secretion has not been reported in the wider literature when investigating responses to PEEK. Here, it is shown that IL-8, CCL2, and CCL4 have significantly positively correlated changes to gene expression and protein secretion upon an increase in dosage of PEEK particles. It is likely that these proteins are not undergoing levels of notable post-translational modification and that folding and trafficking of the proteins upon increases in gene expression are minimal. CCL3, on the other hand, did not have a significant positive correlation and this may be in part due to the negligible differences in both protein secretion and gene expression following the 0.5 and 5 μm^3^ per cell dosages of PEEK. This creates a clustering effect as can be seen in Figure 5. Further investigations could aim to use more intermediate dosages between 5 and 50 μm^3^ per cell PEEK particles in order to draw out a clearer understanding between the clusters observed in this analysis.

Other biomaterials including cobalt-chromium and ceramic oxide nanopowders have been shown to elicit similar responses to the observed response to PEEK particles in this study which can then be ameliorated through TLR4 blockade (Jamieson et al., 2023; Lawrence et al., 2016b). CLI-095 was used as a TLR4 inhibitor as it has previously been optimised for other materials and metal ions in the literature (Lawrence et al., 2016a). IL-8 protein secretion from the THP-1 cells treated with 50 μm^3^ PEEK showed no change with the addition of CLI-095 pre-treatment. This is particularly interesting as previous research has shown a clear link between biomaterials and TLR4 signalling, however, it can be concluded that PEEK does not mediate inflammation from macrophages in the same way as cobalt and ceramics. Therefore, it is likely that another mechanism or pathway is pre-dominant in the activation of macrophage cells to a pro-inflammatory phenotype following exposure to PEEK. Interestingly polyethylene has been shown to significantly increase the production of reactive oxygen species in macrophages (Gazzano et al., 2011) which can lead to pro-inflammatory outcomes. Although TLR4 inhibition did not ameliorate the pro-inflammatory cytokine production in PEEK-treated cells, future studies could investigate the role of other TLRs which have been implicated in biomaterial responses such as TLR2 (McKiel et al., 2023). Investigation of other immune mechanisms or TLRs would greatly strengthen the wider understanding of how polymer biomaterials elicit inflammatory responses *in vivo*.

ROS production increased in a dose-dependent manner in the THP-1 cells and was significantly increased at the 50 μm^3^ per cell PEEK dosage. This is important as increased oxidative stress on tissue resident macrophages in the peri-implant space can lead to increases in pro-inflammatory chemokine secretion and cellular migration. Other orthopaedic polymers such as ultra-high molecular weight polyethylene (UHMWPE) have been shown to upregulate ROS production in THP-1 cells to a near toxic level (Gazzano et al., 2011). Gazzano et al., demonstrated the ability of UHMWPE to induce nitric oxide production in THP-1 cells, another contributor to oxidative stress and inflammation, which highlights the ability of polymers to induce oxidative stress *in vitro*. High levels of oxidative stress directly linked to UHMWPE wear debris from hip replacements has also been observed in synovial inflammation resulting in the need for revision surgery (Fiorito et al., 2001). Further investigation of the effect of PEEK on ROS production could utilise ROS inhibition using antioxidants such as N-acetylcysteine (NAC) and incorporate time-course kinetics to strengthen the wider understanding of how biomaterials influence oxidative stress in immune cells. For full activation of the inflammasome pathway two signals are required; an initial ‘priming’ signal and a subsequent “activation” signal. As the TLR4 pathway had been ruled out, it was important to determine whether PEEK was acting in a PAMP-like manner and priming the THP-1 cell line. Therefore, THP-1 macrophages were primed with PEEK particles followed by a 1 h exposure to ATP in order to mimic the two signals required for full inflammasome activation. There was a significant increase in IL-1β protein secretion from the THP-1 macrophages which received PEEK and ATP compared to those which were exposed only to PEEK. This demonstrated that PEEK causes inflammation in THP-1 macrophages in a PAMP-like manner which is similar to that of ceramic oxide nanopowders (Jamieson et al., 2021), however, TLR4 is not involved in this process. Interestingly, ROS has been shown to influence the expression of IL-8 and CCL2 in THP-1 cells via NF-κB signalling (Akhter et al., 2021) and this may result in the PAMP-like effect observed in this study as opposed to activation via specific surface receptors. Further confirmation using inhibitors of NF-κB signalling combined with antioxidants could be used in future studies to confirm this. Additionally, to confirm the role of the inflammasome in PEEK-mediated inflammatory responses future experiments could utilise NLRP3 inhibitors such as MCC950 as well as the use of Western blotting for cleaved caspase-1.

The immunogenic effect of cobalt has been well established and studies show significant increases in IL-8 protein secretion from cells treated with cobalt ions with secretions at approximately 1,500 pg/mL (Anjum et al., 2016). Additionally, IL-8 protein secretion from ceramic oxide-treated human macrophage cells is significantly increased to approximately 5,000 pg/mL following exposure to Al_2_O_3_ and ZrO_2_ (Jamieson et al., 2021). Together with the findings presented in this study, there is evidence that suggests PEEK particles have a similar immunogenicity to that of Al_2_O_3_ and ZrO_2_ ceramic oxide nanopowders. Interestingly, the protein secretion of IL-8 across studies appears to be lowest in response to cobalt ion stimulation. Investigations directly comparing biomaterials to one another may help to establish a greater understanding of the inflammatory effect of PEEK by assessing additional markers including those which induce osteolysis such as IL-6 and TNFα.

**FIGURE 7:**
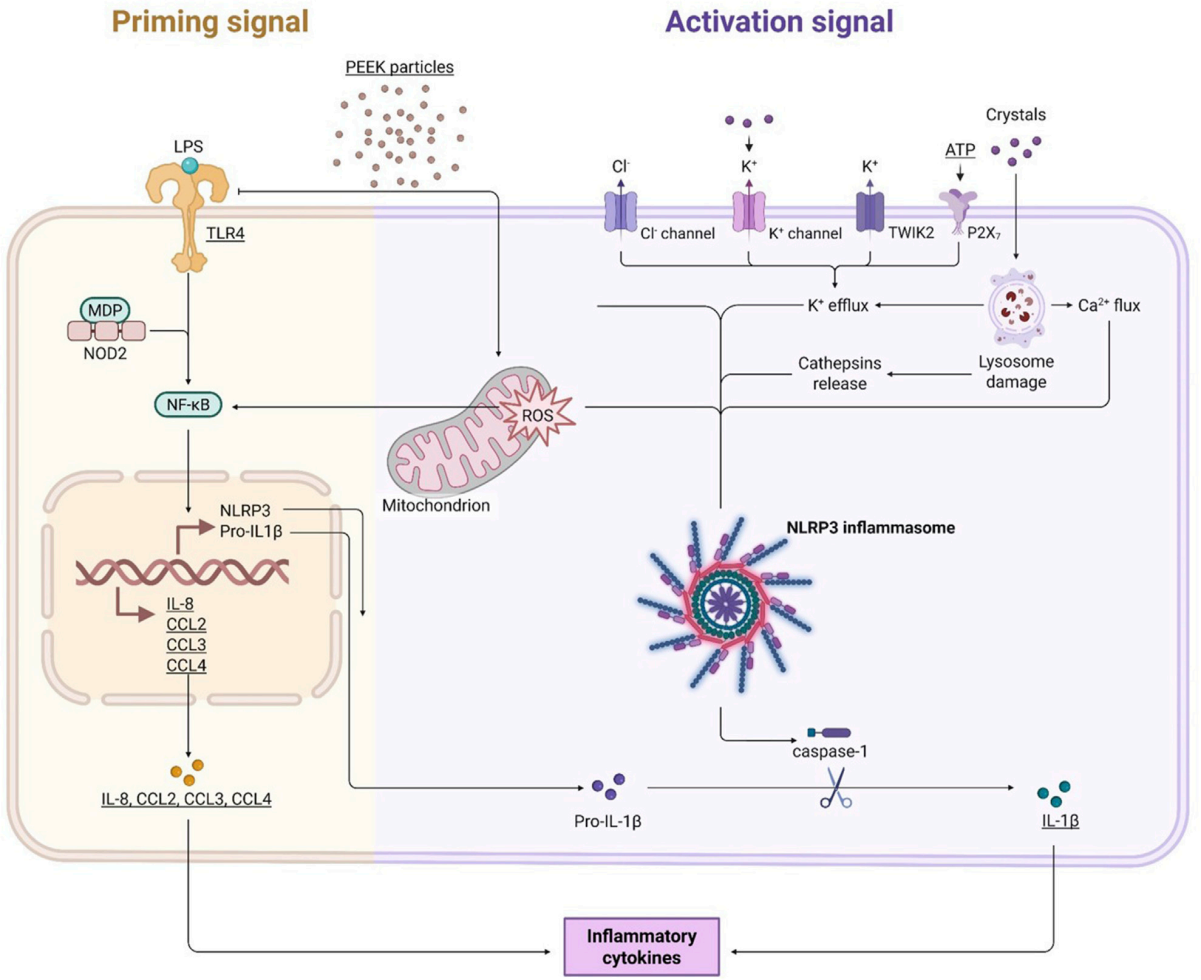
Working model of the inflammatory effects of PEEK particles. PEEK particles cause increases in intracellular ROS levels which can go on to influence activation of both the NLRP3 inflammasome and the NF-κB signalling pathway. Activation of these inflammatory pathways leads to upregulation of pro-inflammatory cytokines IL-8, CCL2, CCL3, and CCL4. IL-1β signal is also increased following a secondary activation signal in the form of ATP exposure. TLR4 is not involved in this process but other TLRs may play a role in the PAMP-like manner by which the PEEK particles are acting.

Overall, based on the work presented in this study it is hypothesised that PEEK particles can induce a pro-inflammatory phenotype in activated THP-1 macrophages as demonstrated by the increases in IL-8, CCL2, CCL3, and CCL4 and that this is likely to be influenced by the production of ROS as well as the NLRP3 inflammasome. A summary of the proposed hypothesis and working model of PEEK-induced inflammation can be seen in Figure 7. Although TLR4 involvement has been ruled out in this study other pathogen recognition receptors such as TLR2 may also mediate the PAMP-like effects observed here. However, further investigation is required to determine the mechanism by which these responses occur and to address caveats to the current investigations. The increase in IL-1β protein secretion observed suggests involvement of the NLRP3 inflammasome and caspase activation, however, using inhibitors of NLRP3 could provide more evidence to support this hypothesis as well as investigating other key components of the inflammasome pathway such as the cleavage of caspase-1. Investigating a larger cytokine and chemokine panel including IL-6, TNFα, and anti-inflammatory IL-10 would allow for a greater understanding of immune responses to PEEK. Additionally, this study only assessed one cell type in the form of an immortalised cell line and therefore the utilisation of multi-cellular experimental designs to assess osteolytic and pro-inflammatory outcomes would help to increase the robustness of these findings. The use of co-cultures which incorporate multiple cell lines, or, the use of primary immune cells from patients with osteoarthritis would allow for additional conclusions regarding the influence of PEEK on the immune system. By moving the current study into a patient-centred model using primary cells there is the potential to strengthen the findings presented here and validate the data *in vivo*. PEEK orthopaedic implants are not currently widely used in the clinic but are being tested in clinical trials for use in hip and knee replacement therefore it is key to understand how these materials interact with the immune system prior to their roll out in the clinic. Due to the lack of clinical data around PEEK wear particles, further investigations should focus on using particle dosages and methodologies similar to that presented in this study before expanding into *in vivo* studies to better understand how patients respond to these implants over time.

## Data Availability

The raw data supporting the conclusions of this article will be made available by the authors, without undue reservation.
