# Supplementary material for: Poly-ether-ether-ketone wear particles induce a pro-inflammatory phenotype in a human monocytic cell line
Source: Front Bioeng Biotechnol. 2025 Aug 5;13:1507248. doi: 10.3389/fbioe.2025.1507248 (PMC12361227; doi:10.3389/fbioe.2025.1507248)
Supplement: Supplementary file 1 [file Supplementaryfile1.docx]

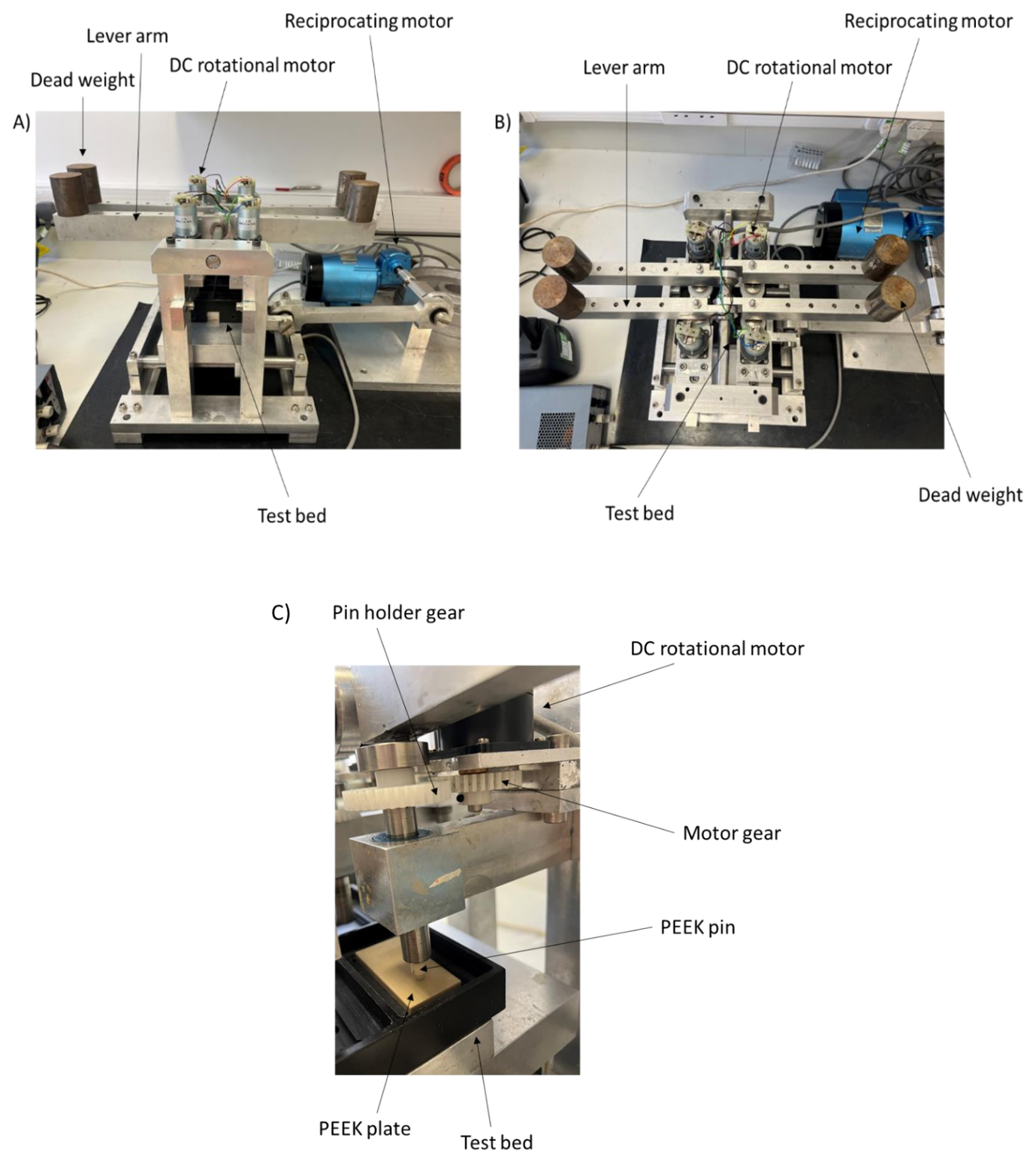
**Supplementary 1: Pin-on-plate wear test simulator set up**

**Supplementary 1:** Set up of the four-station multi-direction pin-on-plate wear simulator used for PEEK particle generation.

**Supplementary 2: PEEK particle charge and zeta potential**

**
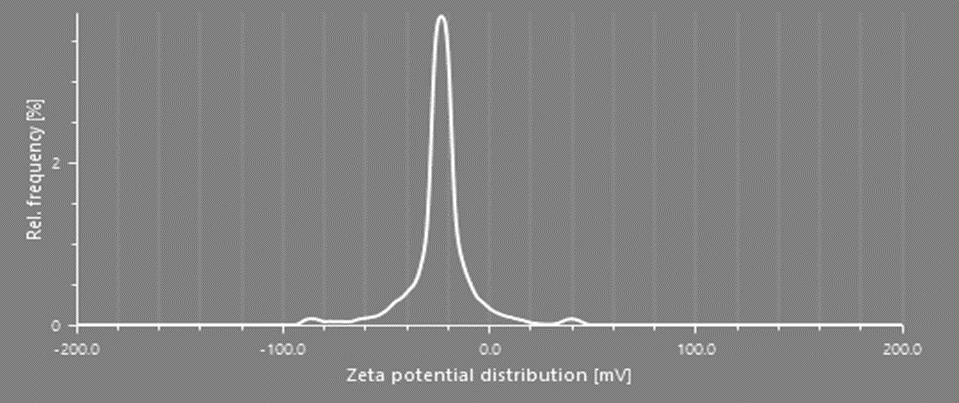
**

**Supplementary 2:** Zeta potential distribution graph of PEEK particles showing a charge of -22.36mV. Graph is representative of three technical repeats.

**Supplementary 3: Housekeeper gene stability upon exposure to PEEK particles**

**Supplementary 3**: House keeper gene stability. Four potential genes were tested prior to RT-qPCR experiments to ensure that a suitable housekeeper gene was used throughout subsequent assays and that inflammatory conditions did not alter expression. There was some variability in the expression level of *HPRT1* and *β-Actin* and therefore they were not considered as suitable housekeeper genes for future experiments. *GAPDH* and *18S* has the most stable average Ct upon exposure to PEEK particles with *18S* maintaining the lowest Ct value of approximately 16 Ct. These findings show that *18S* remains highly expressed with minimal variability in Ct value in THP-1 cells upon exposure to inflammatory stimuli and therefore future RT-qPCR experiments used *18S* as a housekeeper gene.
